# Loss of neuronal population organization links pathology to behavior in a model of Alzheimer’s disease

**DOI:** 10.1073/pnas.2614164123

**Published:** 2026-08-25

**Authors:** Douglas A. Ruff, Drew E. G. Sheets, Ramanujan Srinath, Giovanne B. Diniz, Devon J. Griggs, Danielle Beckman, Sean Ott, Kayla Schwartz, Carissa T. Erices, Scott Muller, Jeffrey H. Kordower, John H. Morrison, Marlene R. Cohen

**Affiliations:** ^a^ Department of Neurobiology, University of Chicago, Chicago, IL 60637; ^b^ California National Primate Research Center, University of California Davis, Davis, CA 95616; ^c^ Arizona State University - Banner Neurodegenerative Disease Research Center, Arizona State University, Tempe, AZ 85281; ^d^ Department of Neurology, School of Medicine, University of California Davis, Sacramento, CA 95825

**Keywords:** population coding, systems neuroscience, Alzheimer’s disease

## Abstract

Alzheimer’s disease and related dementias are typically described at two levels: the accumulation of molecular pathology and the emergence of cognitive impairment. Understanding the relationship between pathology, often studied in animal models, and human cognition will require measurements spanning intermediate scales, including single neurons, neuronal populations, and distributed networks. Here we combine longitudinal measurements of behavior and neuronal population activity with fluid and histological biomarkers in a macaque model of early-stage disease. We find in two animals that visually guided behavior becomes increasingly disorganized, with less consistent and more variable patterns of exploration, despite preserved performance on simple tasks. In parallel, coordinated activity within and between neuronal populations in visual and parietal cortex declines, even as single-neuron tuning and basic feature encoding remain stable. The magnitude of these physiological changes was broadly consistent with biomarker progression. These changes arise when pathology is largely confined to regions providing feedback to visual cortex, indicating that functional disruption extends beyond sites of prominent pathology. Together, these results show that early disease progression is not marked by the loss of individual functions at any single level, but by a selective disruption of coordination across levels, from neuronal populations to behavior. This disorganized state is measurable and modifiable: methylphenidate administration was associated with a transient restoration of behavioral organization. These findings identify disruption of neuronal population organization as a defining feature of early-stage Alzheimer’s disease and establish coordinated population activity as a candidate target for therapeutic intervention.

## Significance

In this study, we use an adeno-associated virus (AAV)-induced longitudinal macaque model of Alzheimer’s disease to examine early changes in behavior, neuronal population activity, and pathology. Our findings suggest that early disease is accompanied by a progressive loss of coordinated population activity. Rather than reflecting a uniform degradation of function, early disease progression is marked by parallel changes in the organization of neuronal populations and behavior. This work extends principles from systems and computational neuroscience to the study of neurodegenerative disease and highlights neuronal population organization as an intermediate level linking molecular pathology to behavior.

Alzheimer’s disease (AD) and related dementias (ADRD) gradually erode the brain’s ability to support flexible, goal-directed behavior, yet the earliest signs of dysfunction are often difficult to detect. Standard cognitive tests typically reveal impairments in executive function and declarative memory only after substantial neuronal loss has already occurred (1–4). Although considerable progress has been made in characterizing molecular pathology, the neural circuit mechanisms linking early pathology to cognitive dysfunction remain poorly understood (5, 6). In addition, many common animal models do not capture the subtle circuit-level disruptions or human-like behavioral changes that precede overt decline. These challenges limit the ability to identify mechanisms and to test interventions at a stage where they may be most effective (7). Addressing this problem requires approaches that link precise, quantitative measurements of behavior to the activity of neuronal populations across the brain.

Visually guided eye movements provide a powerful system for meeting this challenge. During natural vision, human and nonhuman primates typically make four to five eye movements per second, continuously integrating visual information, behavioral goals, and internal states (8–11). These behaviors are rich but highly structured and quantifiable, and decades of basic neuroscience research, particularly in nonhuman primates, have established strong links between neuronal population activity and visually guided behavior (12). Human studies suggest that eye movements are particularly sensitive probes of early AD deficits (13, 14). Finally, the neural underpinnings of flexible exploration and goal-directed eye movements are well studied. They are supported by coordinated activity across multiple brain regions, including early and mid-level visual cortex and association areas involved in attention, decision-making, and working memory (15, 16).

Here, we leverage this well-characterized system to link mechanistic insights and analytic approaches from systems neuroscience to translational questions by longitudinally measuring behavior, neurophysiology, and biomarkers in a macaque model of ADRD (17, 18). Rather than focusing on the loss of individual functions or representations, we examine how the disease progressively alters the organization of behavior and neuronal population activity. We had four goals: first, to identify early changes in the structure of visually guided behavior that emerge before overt impairments; second, to determine how these behavioral changes relate to degradation in the structure of neuronal population activity, despite preserved single-neuron tuning; third, to determine how changes in neuronal population organization relate to markers of disease progression; and fourth, to define population-level signatures of circuit dysfunction that may guide targeted interventions. By linking subtle behavioral disorganization to disruptions in coordinated population activity, this work demonstrates that early disease progression is best characterized as a disorder of organization spanning scales of analysis from molecules to neuronal populations and behavior.

## Results

### Adeno-Associated Virus Injection Induces a Macaque Model of AD.

**Fig. 1.**
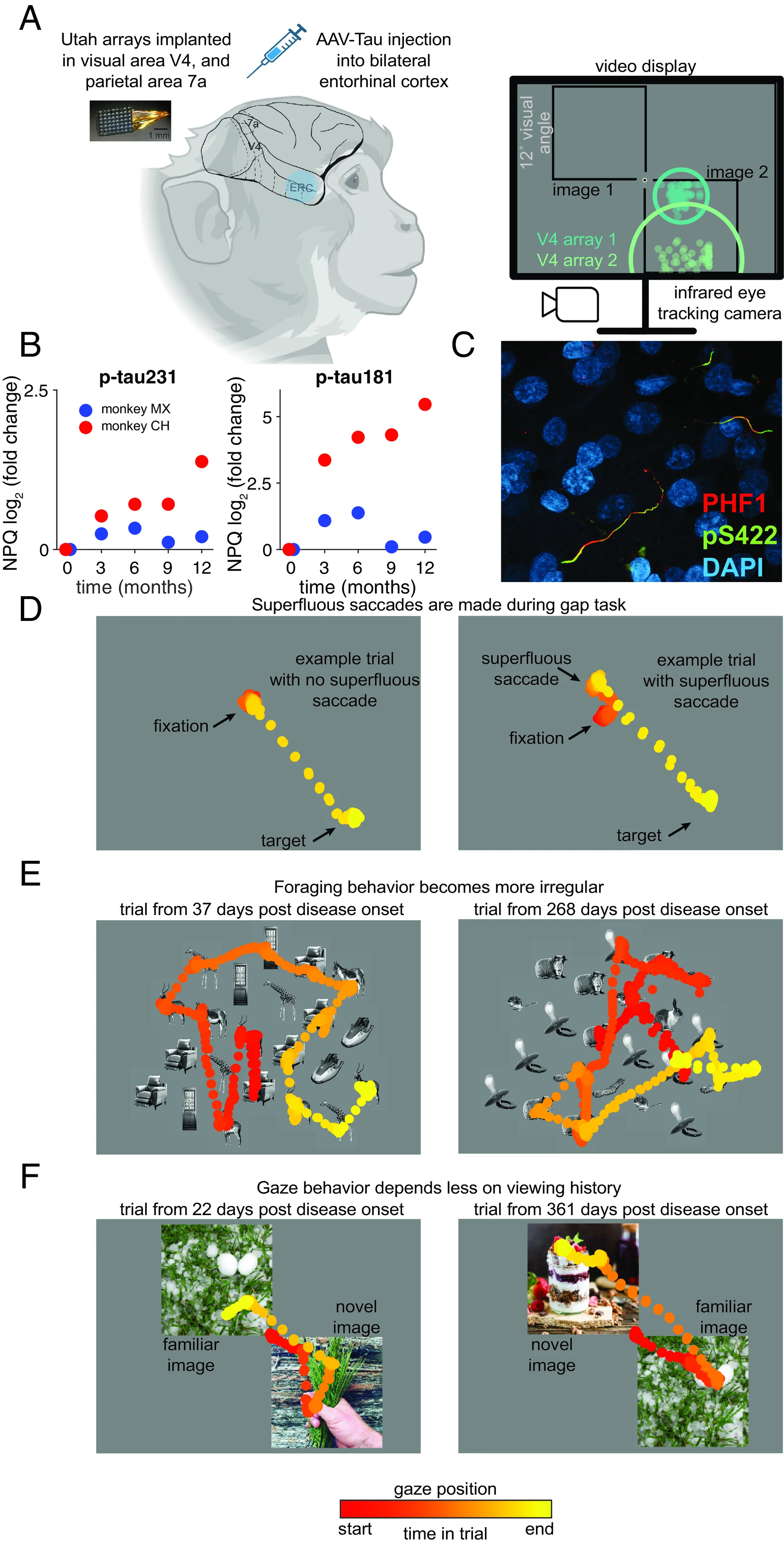
Experimental design and behavioral observations. (*A*) Two monkeys received bilateral AAV-Tau injections into entorhinal cortex to induce progressive tauopathy [*Methods*; (17, 18)]. Three 64-channel microelectrode arrays were chronically implanted: two in visual area V4 and one parietal area 7a. Estimates of the centers of the spatial receptive fields of units from the two V4 arrays relative to fixation from monkey CH are drawn as points on the computer monitor along with an outline of the visual stimulus configuration during the preferential looking task (described below). An estimate of the size of one unit’s receptive field from each array is drawn as a circle. Monkeys performed visually guided tasks based on images presented on a computer screen, controlled by infrared eye tracking, for 1 y following the injections while we simultaneously recorded neuronal responses. (*B*) AAV-induced changes in plasma markers associated with early stages of ADRD were observed in both animals [data are plotted in units of log2 -fold change (20)] and (*C*) histological signs of phosphorylated tau (PHF1, red; pS422, green; overlap, yellow; nuclei, DAPI, blue) were observed in V4. Sparse labeling of phosphorylated tau was seen in the visual and parietal cortex and was predominantly restricted to processes, suggesting that functional changes observed in V4 and 7a do not arise primarily from tau-related cell death in those areas, and implicate synaptic and/or network mechanisms (image from right hemisphere of monkey CH shown, see *SI Appendix*, Fig. S2). (*D*) Two example trials from a gap task where a fixation spot turned off prior to the appearance of a saccade target. On most trials (*Left*), monkeys waited to move their eyes until the appearance of a peripheral target and then made a single saccade to that target. On some trials (*Right*), monkeys made an unnecessary and unrewarded saccade (called a superfluous saccade) prior to the appearance of the peripheral target. (*E*) Two example scan paths during a visual foraging task where fixating certain stimuli (i.e., animals) was rewarded while fixations to others (i.e., objects) were unrewarded. The scan path from the example trial on the *Right* appears more irregular, with larger distances between consecutive fixations and unrewarded returns to previously viewed locations. (*F*) Scan paths from two example trials during a free-viewing preferential looking task. Monkeys were rewarded after accumulating 1 s of total view time on either of the images. In the trial on the *Left*, the monkey looked first and longer at the novel image, explored it, and then switched once to look at the familiar image. On the *Right*, the monkey looked first at the familiar image, and rapidly switched to the novel image. Monkey image in *A* created using BioRender.

The entorhinal cortex (ERC) is among the earliest and most severely affected regions in most human AD patients (19). In our macaque model, pathology is initiated by injecting an adeno-associated virus (AAV) expressing a double human tau mutation (P301L/S320F) into the ERC (Fig. 1*A*). This manipulation produces local tau accumulation and later cell death that spreads gradually to connected regions, with a progression pattern and anatomical specificity that closely resemble human ADRD. The speed of progression is inversely related to the synaptic distance from the ERC, consistent with a spread of pathology that follows corticocortical and hippocampal connectivity (17, 18).

Over the course of disease progression, we measured biomarkers, behavior, and neuronal population responses to obtain a comprehensive picture of the biological, neurophysiological, and behavioral changes. We used blood and cerebrospinal fluid measurements of phosphorylated tau, which are sensitive tools for detecting and staging AD in humans (1, 21), to compare to human disease. In particular, plasma p-tau231 is thought to reflect early disease-related changes (22), and p-tau181 has been shown to predict cognitive decline (23, 24). In our monkeys, both biomarkers evolved as previously reported, confirming induction and spread of tau pathology and supporting the clinical relevance of this macaque model [Fig. 1*B*; (17, 18)]. Because biomarker measurements were collected longitudinally throughout the study, they also provided an opportunity to relate changes in physiology to disease progression.

We performed histological analyses 1 y after disease onset to assess the extent and distribution of pathology in brain areas relevant to this study. In both animals, there were clear signs of abnormally hyperphosphorylated tau throughout the medial temporal lobe (MTL; *SI Appendix*, Fig. S1), consistent with previous reports using this model (17, 18). Tau pathology outside the MTL was detectable but remained limited. In area V4, where the majority of physiological recordings in this study were obtained, tau pathology was observed predominantly in processes, likely belonging to feedback projections (Fig. 1*C* and *SI Appendix*, Fig. S2).

Together, these findings indicate that the disease-related changes observed in V4 should not be interpreted as reflecting lesions at the site of our electrodes. Instead, they are more consistent with alterations to the broader network influencing V4 activity and the visually guided behaviors examined in this study. Because tau pathology was prominent in the MTL and detectable but limited in the extra-MTL cortex, the pattern of pathology is broadly analogous to human Braak stages 2 to 3, which correspond to early to mid-stages of disease (25, 26). This comparison is intended as an approximate anatomical analogy, because there is no established equivalent of human Braak staging for this macaque model. Our results therefore highlight changes that occur during the earliest stages of disease progression.

### Early Behavioral Disorganization Precedes Overt Functional Decline.

The longitudinal design of our study allowed each animal to serve as its own control, providing high sensitivity to detect subtle, progressive changes (2). We trained behavioral tasks before injection of the disease construct and recorded behavioral and neuronal data in daily sessions for 1 y post disease onset. Many aspects of behavior and neuronal responses remained stable: monkeys performed trained tasks effectively, and social, feeding, and general behaviors in the home environment were largely unchanged.

We evaluated visually guided eye movement behaviors in three tasks designed to probe cognitive processes that we hypothesized would change early in disease progression. Despite overall stable performance, we observed rapid, subtle, and consistent changes in how visually guided behaviors were organized.

- Directed saccade (“gap”) task: Early AD in humans is associated with diminished ability to suppress anticipatory or inappropriate saccades, suggesting impaired behavioral inhibition (27). To test for similar deficits, we used a classical “gap task” that manipulates the relative timing between the disappearance of a fixation spot and the appearance of a peripheral target (27). As disease advanced, monkeys became more likely to make unrewarded superfluous eye movements before target onset (Fig. 1*D*). Notably, they continued to generate accurate saccades to targets, indicating a selective decline in inhibitory control rather than a general impairment of oculomotor function.
- Rule-based visual foraging task: Human AD patients show impairments in planning and organizing visual search behavior (14, 28, 29). We assessed these processes using a rule-based foraging task where monkeys were rewarded for fixating only some target images (e.g., animals) and not distractors (e.g., inanimate objects). As disease progressed, monkeys sampled the display more irregularly, consistent with a breakdown in the organization of goal-directed exploration (Fig. 1*E*).
- Preferential looking task: Humans with AD and mild-cognitive impairment and animal models with hippocampal damage have altered gaze patterns during free viewing, particularly showing reduced preference for novel compared to familiar images (30–34). In a preferential looking task inspired by these studies, monkeys displayed reduced preference for fixating novel relative to familiar images (35) and switched their gaze between images more frequently (Fig. 1*F*). These changes parallel those reported early in human AD and in monkeys with hippocampal lesions (30–34, 36–40).

Example trials (Fig. 1) suggest that behavior becomes less organized, less strategic, and faster during disease progression. Because these examples are intended only to illustrate qualitative changes in behavior, we next quantified these observations longitudinally across the entire study. Although many of the individual behaviors shown in Fig. 1 can occur in healthy animals, their frequency and organization changed systematically over time. We quantified these observations as a function of time since disease onset, defining day 0 as the day of AAV-tau injection in the entorhinal cortex. Although overall task performance remained stable, multiple indicators of behavioral organization changed rapidly and systematically.

**Fig. 2.**
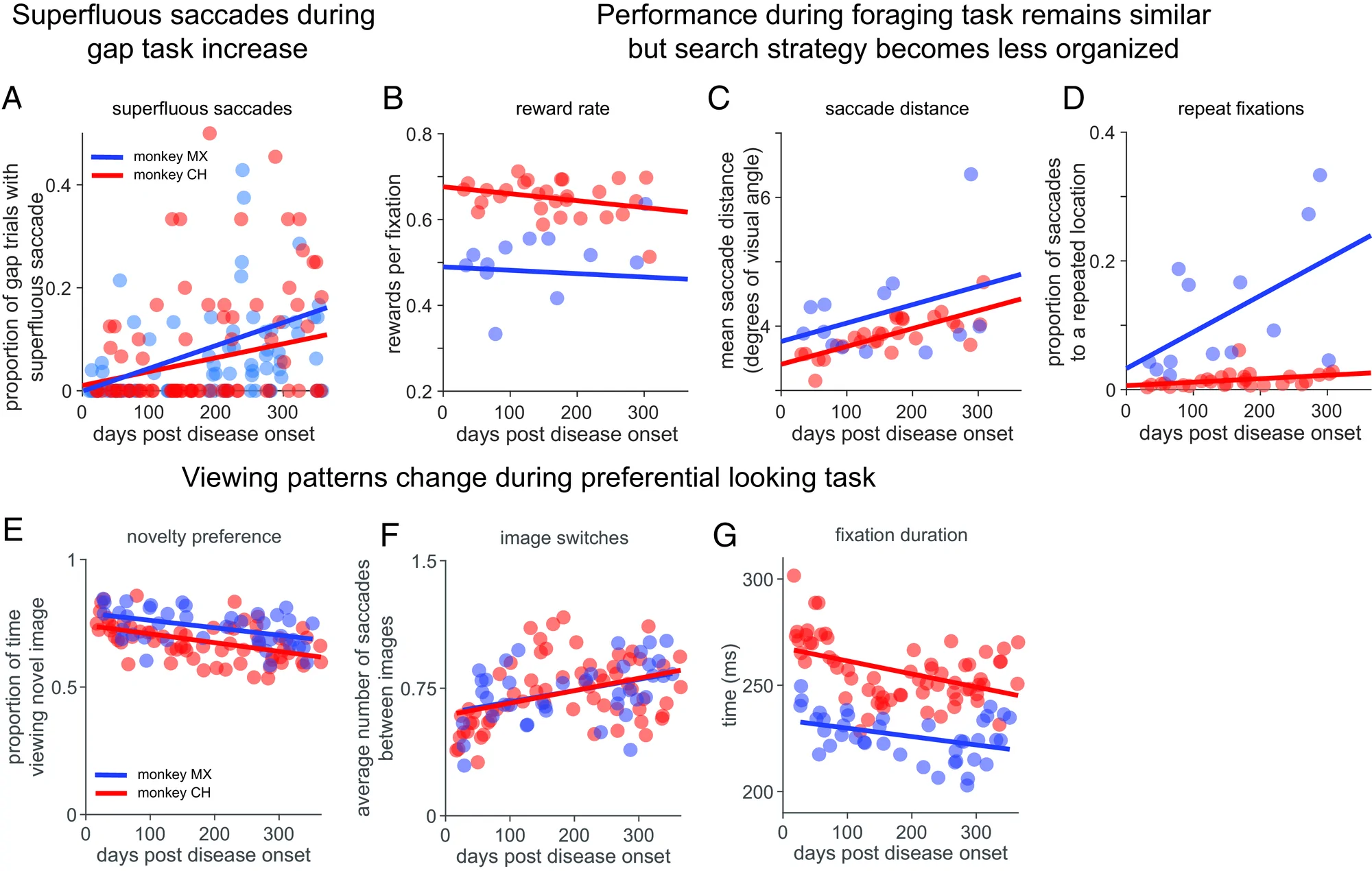
Progressive disorganization of visually guided behavior. (*A*) The frequency of trials in the gap task in which monkeys made an unrewarded superfluous saccade before the target appeared increased across 1 y post disease onset (both slopes *P* < 0.001, all slope tests are F-tests on a linear model against the null hypothesis of a constant slope) (*B*–*D*) Performance during visual foraging task. (*B*) Reward rate per fixation remained largely stable (monkey CH, *P* = 0.12; monkey MX, *P* = 0.8). (*C*) The mean distance between consecutive saccade endpoints increased in one animal, with a similar trend in the other (monkey CH, *P* < 0.001; monkey MX, *P* = 0.21). (*D*) The rate of revisiting a location during a trial, which was always unrewarded, increased (monkey CH, *P* < 0.05; monkey MX, *P* < 0.05). (*E*–*G*) Free viewing during the preferential looking task. (*E*) Preference for viewing novel images decreased (monkey CH, *P* < 0.001; monkey MX, *P* < 0.01). (*F*) The number of saccades that switch between images during a trial increased (monkey CH, *P* < 0.01; monkey MX, *P* < 0.01). (*G*) The amount of time per fixation on the images decreased (monkey CH, *P* < 0.01; monkey MX, *P* < 0.03). Together, these results indicate a gradual loss of structured, goal-directed exploration and reflect early behavioral disorganization.

In the directed saccade task, monkeys continued to generate accurate saccades to peripheral targets. However, they increasingly initiated premature (superfluous) saccades after the fixation point was extinguished but before the appearance of the target (Fig. 2*A*), suggesting a progressive decline in behavioral inhibition, without evidence of more general saccadic impairment.

In the rule-based visual foraging task, monkeys continued to distinguish targets from distractors, and their rates of selecting targets remained relatively stable (Fig. 2*B*). However, their exploration became increasingly disorganized. The average distance between consecutive fixations gradually increased, as did the frequency of revisiting previously sampled locations (which was never rewarded). These metrics indicate increasing variability and reduced structure in goal-directed exploration (Fig. 2 *C* and *D*).

In the preferential looking task, overall engagement with the stimuli remained relatively stable, but viewing patterns changed markedly. The established preference for novel over familiar images declined gradually (Fig. 2*E*). The monkeys also switched their gaze between the two images more frequently (Fig. 2*F*), and the duration of fixations on the images became shorter [Fig. 2*G*; (41–44)]. These changes are consistent with faster, less strategic, and less organized visual exploration.

Together, these results reveal that early ADRD is not defined by failure, but by a progressive degradation of the structure of visually guided behavior. These changes are not easily detectable through coarse or unconstrained behavioral observation but are readily apparent through sensitive, longitudinal measurements.

### Stimulus Representations Remain Intact While Population Structure Deteriorates.

The entorhinal cortex, the site of our AAV-injection, is strongly interconnected with many brain areas, including the ventral visual stream and association areas (45). Given converging evidence for early visual cognitive impairments in human AD (14, 42, 46) and the role of areas like mid-level visual V4 and parietal area 7a in the processes impacted in our behavioral tasks [including visually guided exploration, attention, and visual memory (16, 47–49)], we asked whether early disease progression alters how visual information is represented in those neuronal populations.

Because behavior remained guided by visual information but became disorganized, we hypothesized that neurons in the visual cortex would continue to encode visual features but that the organization of responses across neuronal populations would degrade during disease progression. To test this, we recorded multiunit responses of populations of neurons in area V4 to a range of visual stimuli at various sizes and positions, presented within and outside their classical receptive fields.

**Fig. 3.**
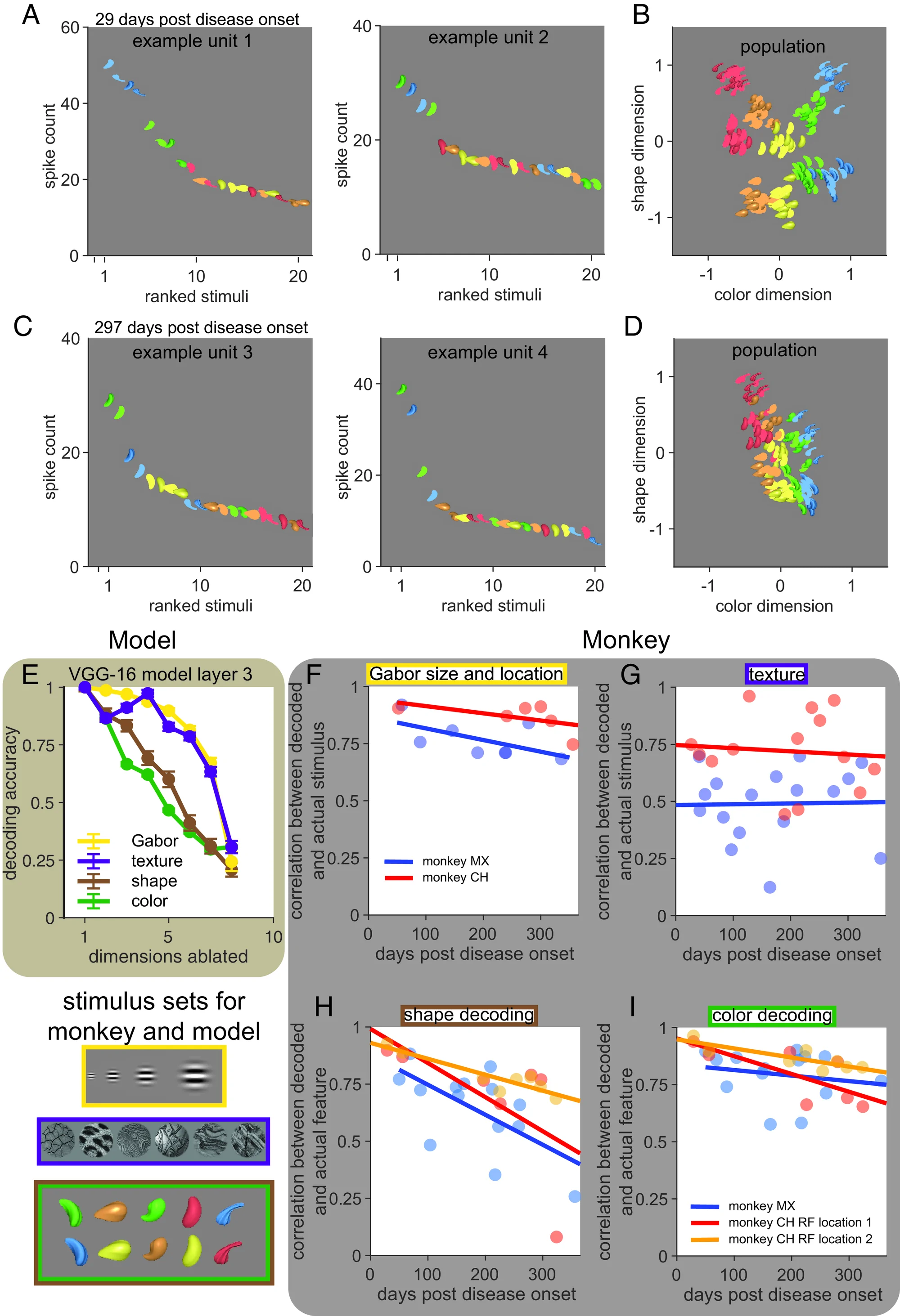
Population-level visual representations lose organization despite stable responses to many visual features. (*A*) Example responses to a set of colored shape stimuli (52) from two example V4 units 29 d post disease onset. Visual stimuli are represented by icons and ordered on the *x*-axis by average response magnitude during a 200 ms window, shifted 50 ms to account for response latency (*y*-axis). (*B*) Projections of V4 population responses from the same example session 29 d post disease onset onto axes in neuronal population space that account for the most color and shape information (identified using QR orthogonalization; see *Methods*). Each icon represents the response of the population to that stimulus on a single trial. (*C* and *D*) Same as *A* and *B* from an experimental session 297 d post onset. While tuning for the stimulus features remained, population responses to different stimuli appear less separable. (*E*) A convolutional neural network model (VGG-16) was used to generate hypotheses about how the disruption of local computations might alter the information about different stimulus features encoded in V4 populations. Removing dimensions of population activity in a V4-like layer of the model more precipitously affected the information represented about our colored shape images compared to texture and Gabor stimuli. (*F*) As predicted by the model, the ability to linearly decode the size and location of a Gabor stimulus from the responses of the recorded V4 neurons remained unchanged for 1 y post disease onset (see *Methods*, monkey CH, *P* = 0.27, monkey MX, *P* = 0.13, all slope tests are F-tests on a linear model against the null hypothesis of a constant slope). (*G*) Similarly, the ability to distinguish between different visual texture stimuli remained unchanged (monkey CH, *P* = 0.76, monkey MX, *P* = 0.93). In contrast, the ability to linearly decode (*H*) shape and (*I*) color information declined post disease onset (two stimulus locations for monkey CH that overlap two clusters of receptive fields, see Methods, shape: monkey CH, *P* < 0.0001 & *P* = 0.13; monkey MX, *P* < 0.01; color: monkey CH: *P* < 0.001 & *P* < 0.05; monkey MX, *P* = 0.51).

Early in disease progression, neuronal populations in V4 exhibited canonical organization for basic features. As in healthy animals, units were tuned for the expected mid-level visual properties [Fig. 3*A*; (50, 51)] and stimulus features that are not typically correlated in natural scenes, such as color and shape, were encoded independently and robustly in the population (52) (Fig. 3*B*). Over time, despite the continued presence of tuned units (Fig. 3*C*), this independence broke down and population responses to different stimuli appeared to become less discriminable (Fig. 3*D*).

The anatomical spread of tau from late to early ventral stream areas that was observed in previous work (17, 18) led us to hypothesize that the representations of features that rely on computations within later or middle ventral areas would degrade earlier than representations of features that rely only on early stages. To assess which features of our stimuli rely on later stages, we selectively removed individual dimensions of the activity of a convolutional neural network (CNN, see *Methods*). CNNs model hierarchical visual processing in the ventral visual stream (53, 54) and allow us to perform “ablation” experiments to assess the impact of different stages of processing on the way different stimulus features are encoded.

Randomly removing dimensions of population activity from a V4-like layer of the model disrupted the encoding of some visual features more than others (Fig. 3*E*). The representation of simple stimulus features (like the spectral components of Gabors, or the discrimination of textures), which are likely inherited from earlier layers, were more robust to ablations in the activity of a V4-like layer than were representations of the shape and color of more complex images (Fig. 3*E*).

Our V4 recordings revealed changes that were broadly consistent with these model predictions. The ability to linearly decode low-level visual features like the position and size of Gabor stimuli or the identity of different visual textures remained relatively stable (Fig. 3 *F* and *G*). This stability also indicates that gradual changes in recording quality did not necessitate a decline in the information available in the recorded population. In contrast, information about the shape and color of complex stimuli declined over time (Fig. 3 *H* and *I*), consistent with a selective degradation of the population structure that supports coordinated encoding of complex features.

Together, these results indicate that early in disease progression, visual feature encoding, especially for features that could be inherited from earlier ventral areas, remains largely intact, even as the structure of population responses degrades. This dissociation reveals that early pathology spares local feature tuning while disrupting signals that organize and coordinate neuronal populations.

### Early Disruption of Signals That Organize Neuronal Populations Accompanies Biomarker Progression.

We therefore asked whether population signatures known to reflect coordinated processing across neurons are disrupted early in disease. Previous work, including from our lab, has demonstrated that the structure of population activity in the visual cortex reflects not only stimulus-driven tuning and patterns of connectivity, but also internal cognitive states like attention, arousal, and task uncertainty (16). These internal states influence the structure and extent to which neuronal responses cofluctuate from trial-to-trial. This covariability is often quantified using pairwise noise correlations [also called spike count correlations or r_SC_, (55)] and by analyzing the geometry of population responses to repeated presentations of the same stimuli (56–58). These population-level signatures are widely interpreted as consequences of local and long-range connectivity, including feedback signals from downstream areas, that shape and organize population responses (59, 60).

In this monkey model, tau pathology was largely confined to medial temporal and connected regions, with little direct accumulation or damage to V4 and 7a at the stages we examined (17, 18). We therefore hypothesized that V4 and 7a population activity would reflect disruption of network effects like feedback and interarea coordination, which could be measurable as disruption of correlated variability consistent with a decline in coordination of population activity. We tested this hypothesis by examining population activity in V4, and 7a during the brief fixation period at the start of each trial, when the screen was blank and stimulus-driven responses were minimal.

**Fig. 4.**
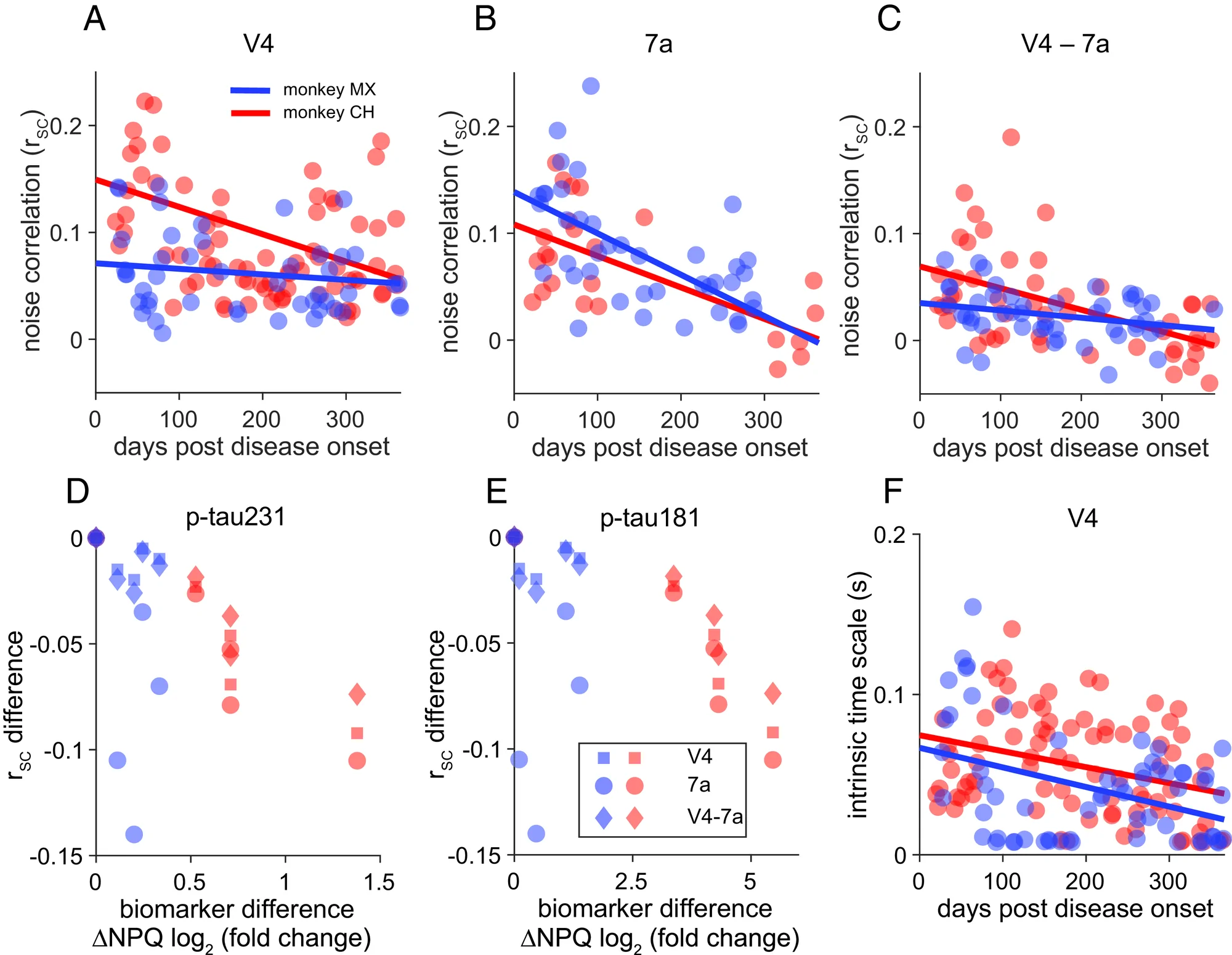
Early disruption of feedback-associated population signals and association with biomarkers. (*A*–*C*) Shared variability decreased during disease progression. The plots show firing-rate matched noise correlations (r_SC,_; see *Methods*) averaged across simultaneously recorded pairs of units measured during a 200 ms period of stable fixation before stimulus presentation. (*A*) Mean shared variability from both monkeys decreased in V4 (monkey CH, *P* < 0.001, monkey MX, *P* = 0.38, all slope tests are F-tests on a linear model against the null hypothesis of a constant slope) (*B*) 7a (monkey CH, *P* < 0.01, monkey MX, *P* < 0.001) and (*C*) between V4 and 7a neurons (monkey CH, *P* < 0.001, monkey MX, *P* < 0.05). (*D* and *E*) Relationship between longitudinal changes in plasma biomarkers and changes in shared variability. Plasma p-tau181 (*D*) and p-tau231 (*E*) values from each animal (Fig. 1*B*) were normalized to predisease levels. Changes in biomarker levels (*x*-axis) were compared to changes in firing-rate matched noise correlations (*y*-axis; same values as in *A*–*C*) estimated at matched time points from the fits in panels *A*–*C*. Each point corresponds to one area (V4, 7a, or V4-7a) from one animal. Negative values indicate reductions in shared variability relative to predisease levels. Larger increases in plasma p-tau181 and p-tau231 were generally associated with larger reductions in shared variability. (*F*) The decay time of V4 autocorrelations (average intrinsic time scale) decreased throughout disease progression, suggesting altered circuit properties (see *Methods*; monkey CH, *P* < 0.005, monkey MX, *P* < 0.001).

Consistent with our hypothesis, we observed an early and progressive decline in these population signatures. During disease progression, correlated variability decreased both within each area and between V4 and 7a (Fig. 4 *A*–*C*). Because these physiological measurements were obtained nearly every day throughout disease progression, they provided the highest temporal resolution measurements in the study and enabled us to relate changes in coordinated activity to evolving biomarker levels. Across areas and animals, larger increases in p-tau181 and p-tau231 were generally associated with larger reductions in correlated variability (Fig. 4 *D* and *E*), suggesting that differences in the magnitude of physiological effects across animals reflect differences in disease progression rather than simple experimental variability. This relationship was particularly apparent in V4 and V4–7a interactions and less so for area 7a in monkey MX, where estimates of correlated variability varied substantially across sessions. In parallel, estimates of intrinsic timescale, which quantify how strongly past neuronal activity influences current response dynamics, also declined [Fig. 4*F*; which uses a method for quantifying timescale that appears in a recent preprint (61)].

Although the relationships between firing rate, correlation structure, and dimensionality are complex and nonlinear (62–65), the pattern we observe indicates a progressive reduction in coordinated population activity. Neuronal firing rates fluctuate more independently of one another, consistent with a gradual disruption of feedback from higher-order areas and/or recurrent interactions within each area. The relationship between these physiological changes and biomarker progression further suggests that neuronal population organization provides an intermediate level linking molecular pathology to behavior. These findings link the preserved stimulus encoding to the early behavioral disorganization we observed in this model of ADRD.

### Proof of Concept: Behavioral Organization Is Partially Modifiable.

The results above reveal a progressive loss of organization in neuronal population activity, even as representations of visual features likely inherited from earlier stages of the ventral visual stream remain relatively preserved. This dissociation indicates that early disease progression disrupts the coordination of neuronal responses before it produces broad degradation of sensory representations. These population-level changes parallel the subtle but consistent disorganization observed in visually guided behavior.

Previous systems neuroscience studies show that correlated variability is modulated by cognitive processes like attention, arousal, or task engagement (16, 66–68). We have also previously shown that methylphenidate (Ritalin), a psychostimulant commonly prescribed for attention deficit hyperactivity disorder, enhances visually guided behavior and modulates correlated variability in the visual cortex (69). Some studies suggest that methylphenidate can reduce apathy in humans with AD (70–75), but its effects on the organization of visually guided behaviors are largely unknown. On this basis, we asked whether the disorganized state we observed during disease progression might be modifiable using the same methods that change neuronal population signatures associated with organized behavior in healthy animals.

To test this possibility, we administered methylphenidate [3.5 mg/kg, an orally administered dose that we previously showed alters behavior and neuronal population activity in the V4 of healthy monkeys; (69)] beginning approximately 6 mo after disease onset, when behavioral disorganization was measurable in both animals (*Methods*). In monkey CH, the drug was administered across 18 d during three-plus weeks of testing starting on day 176, while in monkey MX it was given across 5 d during 1 wk of testing starting on day 174. Monkey CH performed visually guided tasks during experimental sessions that immediately followed drug administration, while monkey MX declined to perform any task.

**Fig. 5.**
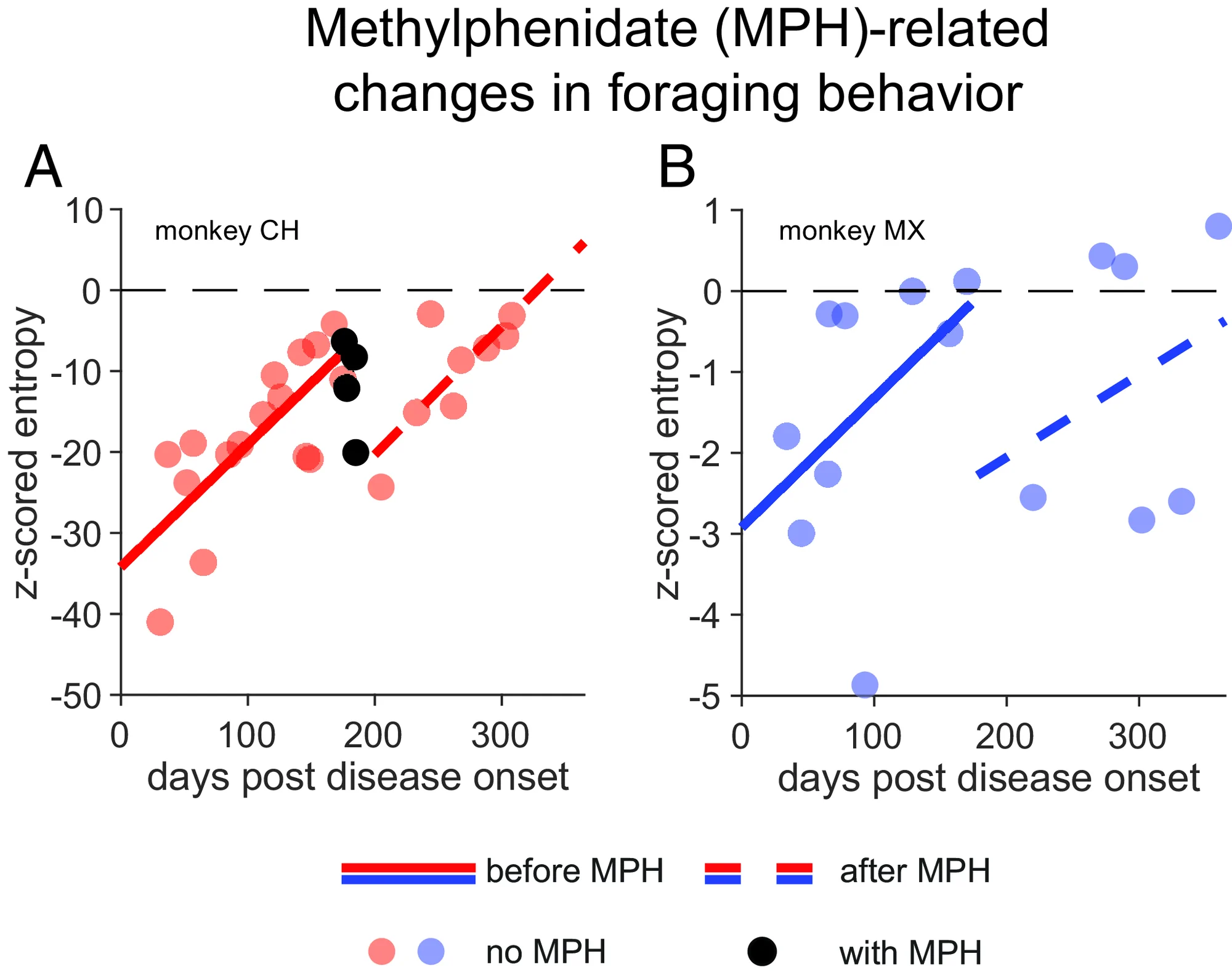
Proof of concept suggesting that principles of systems neuroscience might inspire therapeutic strategy (*A* and *B*) Methylphenidate (administered orally) was associated with a change in behavioral strategy (quantified as z-scored foraging entropy; see *Methods*) during the visual foraging task. In monkey CH, entropy values during methylphenidate sessions (black points) were lower than expected based on the pretreatment trajectory (resampling test comparing observed values to matched-day predictions from the pretreatment model, *P* = 0.03). After treatment ended, entropy resumed increasing at a rate similar to before treatment (slopes of best fit lines before and after methylphenidate administration were not significantly different; monkey CH, *P* = 0.92; monkey MX, *P* = 0.78), but with significantly different intercepts (permutation tests comparing intercepts, *P* < 0.01 for both animals).

In both animals, methylphenidate administration was associated with a partial restoration of organized visual foraging. Before treatment, the entropy of visual search patterns increased steadily over the first 6 mo, reflecting a progressive loss of organized exploration. Following the onset of methylphenidate administration, entropy decreased sharply, returning to levels observed several months earlier. In monkey CH, which performed the visual foraging task during the treatment period, entropy values during methylphenidate sessions were lower than expected from the pretreatment trajectory (resampling test comparing observed entropy to matched-day predictions from the pretreatment model, *P* = 0.03). Once we stopped treatment, entropy did not return immediately to pretreatment levels, even though the direct impacts of methylphenidate should have dissipated within 1 d (69, 76, 77). Instead, entropy began to slowly increase at a rate similar to pretreatment (Fig. 5 *A* and *B*; (slopes of best fit lines are not distinguishable pre vs. post MPH administration; monkey CH, *P* = 0.92; monkey MX, *P* = 0.78; but the intercepts are different; permutation tests, *P* < 0.01 for both animals).

Although this preliminary intervention was not designed as a systematic evaluation of therapeutic efficacy, it provides two insights. First, organized behavior remains at least partially modifiable even in the presence of ongoing pathology. Second, understanding how coordinated neuronal population activity supports behavior may provide a framework for identifying and testing candidate interventions that target circuit-level dysfunction.

## Discussion

We show that early-stage pathology in a model of ADRD is characterized by disruption of neuronal population organization and parallel disorganization of behavior, even as single-neuron tuning and basic sensory encoding remain largely intact. These changes arose at stages when tau pathology in the visual cortex remained limited, indicating that functional disruption extends beyond areas of prominent pathology. Coordinated population activity within and between visual and parietal cortices progressively declined as pathology advanced, and these changes were reflected in structured alterations in visually guided behavior. Together, these findings identify disruption of neuronal population organization as a defining feature of early-stage ADRD and suggest that early dysfunction reflects altered coordination across distributed cortical networks rather than degradation of sensory encoding.

Coordinated neuronal population activity organizes how information is combined across brain areas to guide behavior. When neurons fluctuate together in structured ways and cortical areas interact coherently, distributed representations can be integrated into stable, goal-directed actions rather than fragmented or inconsistent responses. The progressive reduction in coordinated population activity observed here therefore provides a systems-level explanation for why behavior becomes disorganized despite preserved single-neuron tuning (15, 78–80). Early-stage pathology does not primarily eliminate sensory information; instead, it appears to disrupt the coordinated interactions that transform intact representations into organized behavior.

**Fig. 6.**
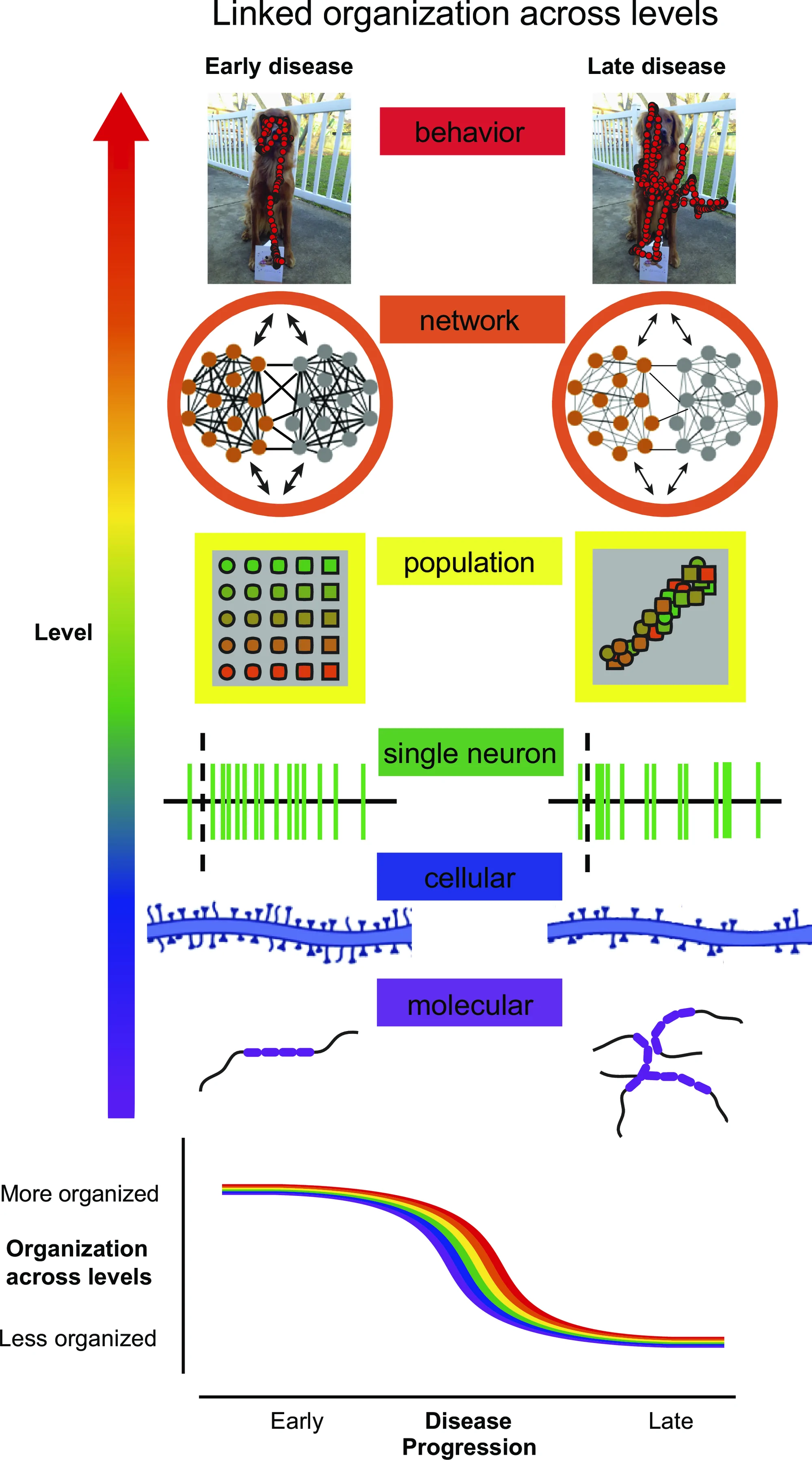
Loss of organization across levels links pathology to behavior. Organized behavior depends on coordinated structure across levels of the brain, from molecules to networks. In the healthy state, molecular organization (tau protein folding), cellular organization (intact synaptic connectivity), single-neuron activity (structured variability and intrinsic dynamics), population representations (separable feature encoding), and network interactions (coordinated activity within and across areas) together support goal-directed behavior (low entropy, structured, efficient). During disease progression, organization at each of these levels is disrupted: tau misfolding and accumulation, synaptic alterations, changes in intrinsic dynamics, reduced separability of population responses, and weakened interareal coordination. These changes emerge in parallel with increasing behavioral disorganization (higher entropy, less structured exploration). This schematic illustrates that disease progression reflects a progressive loss of coordinated organization across levels rather than a uniform loss of function. Molecular and cellular images were created using BioRender.

Early-stage ADRD has traditionally been conceptualized in terms of accumulating molecular pathology and progressive neuronal degeneration. While these processes are central to disease progression, established links between pathology and the cognitively complex behaviors affected in ADRD remain limited. A substantial body of work in systems neuroscience shows that coordinated neuronal population activity provides the substrate for organized, goal-directed behavior. Guided by these principles, we examined whether disruption of population organization could account for early behavioral changes in ADRD. By demonstrating that coordinated population activity declines early in this primate model, even when tau pathology in the visual cortex remains limited and single-neuron encoding is preserved, we provide a causal test of these systems-level ideas in a disease context. Collectively, these findings support a view of early-stage ADRD as a disorder of organization that manifests in parallel across molecular, physiological, and behavioral domains (Fig. 6). Neuronal population organization thus emerges as a central characteristic of early-stage ADRD that remains, at least in part, modifiable.

### Disorganization of Coordinated Population Activity Underlies Early Cognitive Dysfunction.

The earliest changes we observed during disease progression were not impairments in basic task performance or worsening of single-neuron tuning, but alterations in behavioral structure and strategy. Early in disease progression, monkeys continued to perform the basic visual tasks, but their behavior became progressively less structured, less strategic, and faster. These behavioral changes were mirrored at the population level: while representations of visual features likely inherited from earlier ventral stream areas remained relatively preserved, the coordination of neuronal responses deteriorated. Population signatures that, in healthy animals are modulated by attention, arousal, or task uncertainty (16, 66–68, 81, 82) declined early in disease progression, suggesting that cognitive symptoms may emerge from disruptions of the same network computations that support flexible behavior in healthy individuals.

By directly measuring changes in the activity of neuronal populations and quantifying the magnitude and structure of correlated variability, we show that the organization of population activity is altered early in disease progression in ways that are not readily apparent from single-neuron responses or coarse behavioral observation. Because these signatures are thought to depend on feedback and corticocortical interactions that support flexible behavior, their early disruption implicates those mechanisms and is consistent with the notion that corticocortical circuits interconnecting association regions are highly vulnerable in ADRD (83).

Longitudinal measurements of plasma biomarkers provided an additional link between molecular pathology and neuronal population activity. Across animals and recording sites, larger changes in p-tau181 and p-tau231 were generally associated with larger reductions in correlated variability, suggesting that differences in the magnitude of physiological effects reflect differences in disease progression rather than simple experimental variability. Although these biomarkers provide only a coarse measure of pathology, these findings support the idea that neuronal population organization represents an intermediate level linking molecular changes to behavior.

### The Degradation of Behavioral Organization Is Partially Modifiable.

Because we observed that coordinated behavior and the coordinated neuronal population activity that underlies it deteriorates during disease progression, we asked whether this degraded state could be rescued. Previous work in systems neuroscience demonstrates that coordinated neuronal population activity is dynamically modulated by cognitive processes like attention, arousal, and learning (16, 66–68, 81, 82). We previously showed that methylphenidate can alter coordinated population activity and behavior in ways that resemble successful attention and arousal in healthy animals (69). On this basis, we conducted a preliminary test of whether the disorganization arising during neurodegeneration could be partially reversed.

We found that methylphenidate was associated with a temporary restoration of behavioral organization in this model of early-stage ADRD. Although this preliminary demonstration was not designed as a systematic assessment of pharmacological efficacy, it indicates that the degradation of organized behavior and its neural underpinnings remain partially modifiable, at least in the early stages. These findings illustrate how principles and measurements from systems neuroscience can generate and evaluate candidate interventions targeting circuit- and computation-level dysfunction.

These results also suggest that key features of disease progression resemble the behavior of healthy individuals under high cognitive load. This suggests that, at least in early stages, the system is not operating in a fundamentally different mode, but instead closer to its limits. In healthy conditions, behavior is typically organized but can become disorganized under heavy load. In early ADRD, this transition appears to occur at lower levels of demand, reflecting a shift toward a less organized state. Consistent with this interpretation, our methylphenidate results suggest that aspects of this disrupted structure can be transiently restored.

### Longitudinal Primate Models Link Pathology to Behavior.

This work underscores the potential of longitudinal nonhuman primate models for studying early-stage neurodegenerative disease (84). Longitudinal monitoring of behavior and large-scale neuronal population activity in the same individuals makes it possible to track disease progression, identify early biomarkers, causally test hypotheses, and evaluate potential interventions in a controlled yet biologically relevant system. The similarity of monkey and human brains and behaviors enhances the translational potential of these approaches and findings. Collectively, these approaches enable investigation of intermediate population-level changes that link molecular pathology to complex behavior in ways that are difficult to achieve in other model systems.

### Conclusion and Impact.

Taken together, our findings suggest that early-stage ADRD progression is best understood as a progressive loss of organization that unfolds in parallel across levels of analysis (Fig. 6). Despite preserved single-neuron tuning and intact encoding of many basic visual features, we observe coordinated changes in the structure of neuronal population activity, interarea interactions, and visually guided behavior that accompany well-known changes in molecular and cellular structure. Together, these observations suggest that pathology does not simply eliminate representations, but disrupts the coordinated organization that enables distributed activity to support consistent, goal-directed behavior. This perspective places disease progression along a continuum from cognitive resilience to cognitive disorganization and identifies organization as a property that links molecular pathology, population activity, network coordination, and behavior. In this view, neuronal population organization is a central substrate of cognitive integrity, and its degradation provides a mechanistic bridge between pathology and behavior.

## Methods

We measured plasma biomarkers, behavior, and neuronal population activity in two macaque monkeys over the course of 1 y. Animals performed oculomotor tasks while we recorded extracellular spiking activity from chronically implanted electrode arrays in the visual and parietal cortex, and blood samples were collected repeatedly throughout the study. To model Alzheimer’s disease, we delivered an adeno-associated virus expressing a human double tau mutation to bilateral entorhinal cortex, and histological analyses at the conclusion of the study confirmed successful model induction. Full details of experimental procedures and statistical analyses are provided in the *SI Appendix, SI Methods*.

## Supplementary Material

Appendix 01 (PDF)

## Data Availability

Neuronal and behavioral data have been deposited in figshare (10.6084/m9.figshare.32961443) (85).
