# Supplementary material for: Loss of neuronal population organization links pathology to behavior in a model of Alzheimer’s disease
Source: Proc Natl Acad Sci U S A. 2026 Aug 25;123(35):e2614164123. doi: 10.1073/pnas.2614164123 (PMC13534665; doi:10.1073/pnas.2614164123)
Supplement: Supplementary file 1 [file pnas.2614164123.sapp.pdf]

***SI Appendix, SI Methods*:**

Animals

Two adult male rhesus macaques (13 to 15 years old, conforming to the age ranges used in (17, 18)) had bilateral stereotaxic injections of an adeno-associated virus expressing a human double tau mutation (AAV-P301L/S320F, 1.176 × 10ˆ13 genomic copies/mL) into entorhinal cortex (ERC) (17, 18). To increase the area of AAV expression, two injections were made per hemisphere. Injections were performed using the StealthStation surgical navigation system (Medtronic, Minneapolis, MN) using presurgical MRIs as guidance. During the same surgery, and following the AAV injections, we implanted microelectrode arrays (Blackrock Microsystems, 48 electrodes per array, arranged in a 6x8 rectangle) sub-durally in the left hemisphere of each animal. We implanted one array in parietal area 7a and two arrays in visual area V4. The arrays were connected to percutaneous connectors, which allowed electrophysiological recordings. The distance between adjacent electrodes was 400 µm, and each electrode was 1 mm long. All experiments were conducted with approval by the Institutional Animal Care and Use Committee (IACUC) at the University of California–Davis.

AAV preparation

The adenovirus used in this work has been described in detail elsewhere (17, 18, 84). A capsid 1 adenovirus was packaged with one copy of human 0N/4R tau-containing two mutations (P301L/S320F) that render it more aggregation-prone than wild-type tau. The expression of this tau insertion is under the control of the hybrid cytomegalovirus enhancer/chicken β-actin (CMV/CBA) promoter, a CBA intron (first intron of chicken β-actin gene plus the splice acceptor of the rabbit β-globin gene), woodchuck hepatitis virus post-transcriptional regulatory element (WPRE), and bovine polyA. Virus packaging and purification were performed at the Penn Vector Core (University of Pennsylvania), following gold standard laboratory practices, including endotoxin testing (<5 endotoxin units per mL), purity testing (sodium dodecyl sulfate-polyacrylamide gel electrophoresis), and titration (three rounds of digital polymerase chain reaction). After preparation, aliquots of AAVs were stored at −80°C until surgery.

Structural MRI and surgical planning

Pre-surgery structural imaging of both monkeys was performed using a 3T MRI scanner (Skyra, Siemens Healthcare, Germany) with an 8-channel receiver coil optimized for monkey brain scanning (Rapid MRI, Columbus, OH). T_1-weighted images were acquired using the magnetization-prepared rapid acquisition with gradient echo (MPRAGE) pulse sequence with the following parameters: field of view = 154×154 mm; 480 sagittal slices; TR/TE = 2500/3.65 ms; flip angle = 7°; TI = 1100 ms; voxel size 0.6×0.6×0.6 mm, interpolated to a resolution of 0.3×0.3×0.3 mm. Anatomical images were used to identify the location of ERC, and, in conjunction with fiducial markers, determine appropriate injection sites.

CSF, serum, and plasma collection and processing

Cerebrospinal fluid (CSF), plasma, and serum were collected before the initial surgery, approximately once per month post-surgery, and immediately before euthanasia. Before each collection, animals were sedated with ketamine (5–30 mg/kg, intramuscular) and dexmedetomidine (0.0075–0.015 mg/kg, intramuscular). To collect CSF, a 23-gauge spinal needle was inserted into the subarachnoid space of the cisterna magna, and 1–2 mL of CSF was aspirated into sterile glass vials, which were then centrifuged at 400×*g* for 10 minutes at 4 °C to remove cellular debris. The supernatant was then aliquoted into low protein-binding cryotubes

and stored at –80°C. To collect serum and plasma, ≈30 mL of venous blood was collected in EDTA-containing tubes (plasma only) and centrifuged at 1500×*g* for 15 min. The samples were divided into 0.5 mL aliquots and stored at −80°C.

Phosphorylated tau quantification in biofluids

Plasma and CSF samples were analyzed using the NULISAseq™ CNS Disease Panel 120 (Alamar Biosciences, Fremont, CA), a sequencing-based fluid proteomics platform with increased sensitivity and dynamic range compared to traditional quantification techniques (83). Briefly, specimens were assayed in singlets in the same plate to minimize batch effects. Each assay plate included multiple internal and external controls: three sample controls (derived from a well-characterized plasma source) and four negative control wells (buffer only, used to establish the limit of detection for each analyte), as well as internal controls (IC) spiked into every well to compensate for technical variability.

All assay steps were executed automatically using a preprogrammed ARGO HT system (Alamar Biosciences). Pooled sequencing libraries were generated and sequenced on an AVITI benchtop sequencer (Element Biosciences, San Diego, CA). Raw sequencing counts for each analyte were normalized to the corresponding IC counts and log2-transformed, yielding results in normalized protein quantification (NPQ) units. Scaling, normalization, and log2 transformation were performed automatically by the ARGO Control Center (ACC) software (Alamar Biosciences) based on the FASTA file generated by the sequencer.

Sample integrity was evaluated using three criteria: number of IC reads (> 1,000 reads), total number of reads (> 500,000), and sample IC reads relative to the plate median (+-40% of the plate median). All samples included in this study cleared all three criteria (IC reads min = 10,565; total reads min = 1,457,005 reads; IC% max = 22.56%). For the two targets reported in this study, pTau-181 and pTau-231, all samples were above the limit of detection. Intraplate coefficient of variation (CV%) was 11.37% for pTau-181 and 6.09% for pTau-231.

Animal observation and health monitoring

The weights of both animals were tracked weekly and remained stable, with both slightly increasing across the year of testing. Both animals routinely received physical examinations as well as close observation of food intake and informal assessments of cage-side behavior. Throughout the year of testing post disease onset, both animals maintained stable relationships with their pair-housed partner and frequently engaged in routine social interactions and grooming bouts. Neither animal exhibited major behavioral changes that warranted external intervention. Further, throughout the year of testing, both animals remained willing to enter their chair and work for juice and food rewards in the experimental testing rig. The animal controlled the duration of each experimental session (a session ended when they lost interest in doing the task).

Behavioral training and testing

During training and recording sessions, the monkeys sat facing a computer screen in a primate chair that provided access to a rigid straw that was used to deliver liquid rewards. During training and some testing sessions, padded spacers were placed near, but not touching, the sides of the head to encourage a forward-facing sitting position. In both animals, these were eventually abandoned during recording sessions. Eye tracking was used to control the behavioral task and

successful tracking was possible when monkeys faced forward and placed their mouths on the reward straw. Otherwise, behavioral trials did not initiate. Monkeys were given *ad libitum* access to water in their home enclosure and were rewarded with juice and food rewards during training and testing. They received the remainder of their food rations after the completion of their daily testing.

The monkeys were trained for between 3 and 6 months prior to surgery to perform a variety of visually guided behavioral tasks. For all tasks, each trial began when the monkey fixated a small spot in a 1.0° circular fixation window in the center of a video display for 200-250ms. The color of the fixation spot was linked to the different tasks, but the monkeys only ever performed one task at a time, in blocks of trials that typically lasted for an entire experimental session.

This manuscript discusses three classes of behavioral tasks. The directed saccade ("gap") task (Figure 1D), required the monkeys to maintain fixation on a central spot until a peripheral reward target appeared on the screen at one of four possible locations. After target onset, the monkey had to saccade to the target within 400ms to receive a liquid reward. During the initial fixation period, visual stimuli were displayed for either 200 or 300ms to measure response properties of the recorded visual neurons. A variety of stimuli were used, some of which are reported in Figure 3. Inspired by past studies using this task to investigate the ability to withdraw fixation and attend to peripheral targets (26), we varied the relative timing of fixation offset and target onset. The peripheral target could appear while the fixation spot was still on the screen, at the same time the fixation spot disappeared, or following the offset of the fixation spot. In all cases, the monkey was rewarded for making a saccade to the target within 400ms of its appearance on the screen.

In the rule-based visual foraging task (Figure 1E), the monkey fixated a central spot, and then an array of visual stimuli was displayed. After the offset of the fixation point, the monkeys were rewarded for fixating stimuli from a target category (e.g. animals) and not for fixating distractor stimuli (e.g. inanimate objects). Monkeys were rewarded the first time they fixated inside an invisible circle around a target stimulus and were not rewarded for repeatedly fixating the same target location or for fixating a distractor. The position of target and distractor stimuli changed on every trial and different target and distractor stimuli were used in different experimental sessions.

In the preferential looking task (Figure 1F; (30, 33)), after the monkey fixated a central spot we displayed two visual stimuli in opposite hemifields. After 250ms, the fixation spot turned off. This indicated that the monkey could freely view the stimuli. The monkeys were rewarded for accumulating 1000 ms of total viewing time on either or both images. Visual stimuli were pseudo-randomly selected from the THINGS image set (85) and the amount of visual exposure to each image was manipulated such that stimuli had either never been viewed before (novel) or had been previously viewed anywhere from once to many hundreds of times (familiar).

Neuronal recordings

We recorded neuronal activity from the electrode arrays during daily experimental sessions for one year in each animal. Using our recording methods, it is nearly impossible to tell whether we recorded from the same single– or multi–unit clusters on subsequent days. To be conservative,

the analyses presented here are performed on data from single recording sessions and then, when relevant, are compared across time.

An experimental session was included in neuronal analyses (Figures 3 and 4) if the monkey completed at least 35 behavioral trials (monkey CH, mean 62 trials; monkey MX, mean 45 trials) and a sufficient number of units responded at 10% more to the visual stimulus or target onset than when the monkey fixated a blank screen at the beginning of each trial. In Figure 4, the minimum was 45 active V4 units (monkey CH, mean 104 units, minimum 46 units, maximum 128 units; monkey MX, mean 75 units, minimum 46 units, maximum 122 units), 10 7a units (monkey CH, mean 22 units, minimum 11 units, maximum 38 units; monkey MX, mean 28 units, minimum 13 units, maximum 53 units), and 100 V4-7a unit pairs (monkey CH, mean 1382 pairs, minimum 115 pairs, maximum 4826 pairs; monkey MX, mean 1729 pairs, minimum 128 pairs, maximum 4270 pairs).

To ensure that changes in firing rates cannot explain the noise correlation changes reported in Figure 4 (53, 65, 86), we calculated noise correlations by subsampling units such that there was a matched mean firing rate for all included units across the entire year of testing. Units were randomly dropped from inclusion until the distribution of baseline firing rates had an average between 20 and 30 spikes per second.

Data analysis and statistical comparisons
We assessed the statistical significance of changes in behavioral and neuronal measures across time using F-tests on a linear model against the null hypothesis of a constant slope.
We assessed stimulus information in populations of neurons using linear regression with neurons as regressors, using leave one out cross validation across trials. We quantified decoding performance as the correlation between predicted and true stimulus or visual feature values.

QR orthogonalization
To visualize changes in the structure of population activity (Figure 3B and 3D), we performed PCA on the average population responses to the stimuli shown in Figure 3A and then used linear regression to identify the best dimension for representing each of the two features of interest, color and shape. We next performed QR decomposition on these axes and projected responses into these orthogonalized dimensions. Responses were then scaled by subtracting the mean and dividing by the maximum response from each session so that responses could be compared across sessions.

Intrinsic timescale calculation
To estimate the intrinsic timescale of the activity of each unit, we computed a shuffle corrected, mean-rate normalized, autocorrelogram (Smith and Kohn, 2008) using spike times during a 200 ms window of stable fixation on a blank screen at the beginning of each trial. We then fit an exponential decay function to each autocorrelogram and defined intrinsic timescale as the decay time constant. To account for refractory periods between spikes (59), we began the fitting procedure 3ms after the 0 lag. We included units that were well fit by an exponential function (those with goodness of fit (R^2) > 0.5). This analysis was only performed on V4 data because of an insufficient number of units in 7a whose autocorrelograms were well fit by an exponential function.

Behavioral entropy calculation

To quantify the structure of visual foraging behavior (Figure 4E, F), we computed a transition entropy metric based on sequences of fixations within each trial. For each fixation location, we calculated the proportion of next fixations at each of the other locations. We defined transition entropy as the Shannon entropy of this distribution,

$$H = -\sum_{i,j} p_{ij} \log_2 p_{ij},$$

where p_{ij} denotes the probability of transitioning from location i to location j. Higher entropy values indicate more uniform, less structured transition patterns, whereas lower entropy reflects more stereotyped or organized foraging behavior. To normalize for factors related to the number of fixations within each trial, we generated a null distribution by randomly shuffling the order of fixations within each trial and recomputing entropy. Entropy values were z-scored relative to this shuffled distribution ($z = (H_{\text{actual}} - \mu_{\text{shuffled}})/\sigma_{\text{shuffled}}$), such that negative values indicate more structured behavior than expected by chance, and values near zero indicate approximately random foraging. This metric captures the degree of structure in fixation sequences without imposing assumptions about specific search strategies or saccade statistics. We included sessions in this analysis if there were at least 20 successfully completed trials.

Convolutional neuronal network model

To generate hypotheses about how disease progression might affect stimulus representations, we used activations from a pre-trained convolutional neural network (VGG-16). We measured model responses to our stimuli and used PCA to reduce the dimensionality of responses from the pooling layer neurons of model layer 4. We then decoded stimulus features (Figure 3E) while randomly removing different numbers of dimensions to simulate disease related changes.

Methylphenidate administration

Methylphenidate was orally administered for short bouts starting approximately six months after disease onset. We used a dosage of 3.5 mg/kg based on our previous studies (67). In monkey CH, the drug was administered on 18 days during three-plus weeks of testing starting on day 176, while in monkey MX it was given on 5 days during one week of testing starting on day 174. The methylphenidate was dissolved in 20-30 mL of flavored sugar water and administered orally using a method adapted from (67, 87). As in our previous study, methylphenidate was administered 30-45 min before the start of the experiment (67). An additional 6 day bout of MPH was administered in monkey CH starting on day 251, but an insufficient amount of data were collected during the time spans to expand the fitting performed in Figure 4E.

Perfusion and tissue preparation

Necropsy was performed by a team of trained pathologists in a biosafety level 2 environment. On the day of euthanasia, monkeys were deeply anesthetized with ketamine (25 mg/kg) and transcardially perfused with saline until the vascular bed was cleared, as described previously (17, 18). After perfusion, brains were harvested, hemisected, and sectioned into 6 mm-thick coronal blocks. Blocks were fixed by immersion in 4% paraformaldehyde with 0.125% glutaraldehyde in phosphate buffer pH 7.4 at 4°C for 48 h under agitation. After the washes, blocks were stored in phosphate-buffered saline (PBS) with 0.1% sodium azide at 4°C until processed. Semiseriated, 30 μm-thick coronal sections were obtained by cutting blocks

containing the ERC and HF in a freezing microtome. Sections were stored in antifreeze solution with 0.1% sodium azide at -20°C.

Immunofluorescence and histological staining

We used immunofluorescence to validate the AAV injection sites and expression of tau proteins, based on the protocol in our previous studies (17, 18). Nuclei were stained with 4′,6-diamidino-2-phenylindole (DAPI 0.001 mg/mL; Invitrogen). Neuronal markers NEUN (1:1000; 266 004, Synaptic Systems GmbH, Göttingen, Germany) and Pan-Neurofilament (panNF; 1:400; DMAB 7133, Creative Diagnostics, New York, NY) and tau epitopes AT8 (1:500; MN1020, Invitrogen, Waltham, MA), pS422 (1:1000; ab79415, Abcam), and PHF1 (1:1000, generously provided by Dr. Nicholas Kanaan) were used for validation.

Microscopy, image analysis, and histological validation

All images were acquired using an upright microscope (AxioImager Z2; Carl Zeiss AG, Oberkochen, Germany) equipped with an Axiocam 620 mono camera, LSM800 confocal head, and a 32-channel Airyscan detector (Zeiss). Low-magnification tiles were acquired using single-plane widefield illumination, while high-magnification images were acquired as 3D stacks (20-30μm) and collapsed via maximum-intensity projection for reporting. Images were acquired using the ZEN core software (Zeiss). Brightness and contrast were adjusted in uniform manner across images. To ensure that the model was successfully induced, we identified injection tracks pointing to the entorhinal cortex (ERC), and markers of tau.

Additional references:

84. E. J. Koller *et al.*, Combining P301L and S320F tau variants produces a novel accelerated model of tauopathy. *Human Molecular Genetics* **28**, 3255-3269 (2019).
85. M. N. Hebart *et al.*, THINGS: A database of 1,854 object concepts and more than 26,000 naturalistic object images. *PLOS ONE* **14**, e0223792 (2019).
86. M. M. Churchland *et al.*, Stimulus onset quenches neural variability: a widespread cortical phenomenon. *Nature neuroscience* **13**, 369-378 (2010).
87. P. L. Soto *et al.*, Long-Term Exposure to Oral Methylphenidate or dl-Amphetamine Mixture in Peri-Adolescent Rhesus Monkeys: Effects on Physiology, Behavior, and Dopamine System Development. *Neuropsychopharmacology* **37**, 2566-2579 (2012).

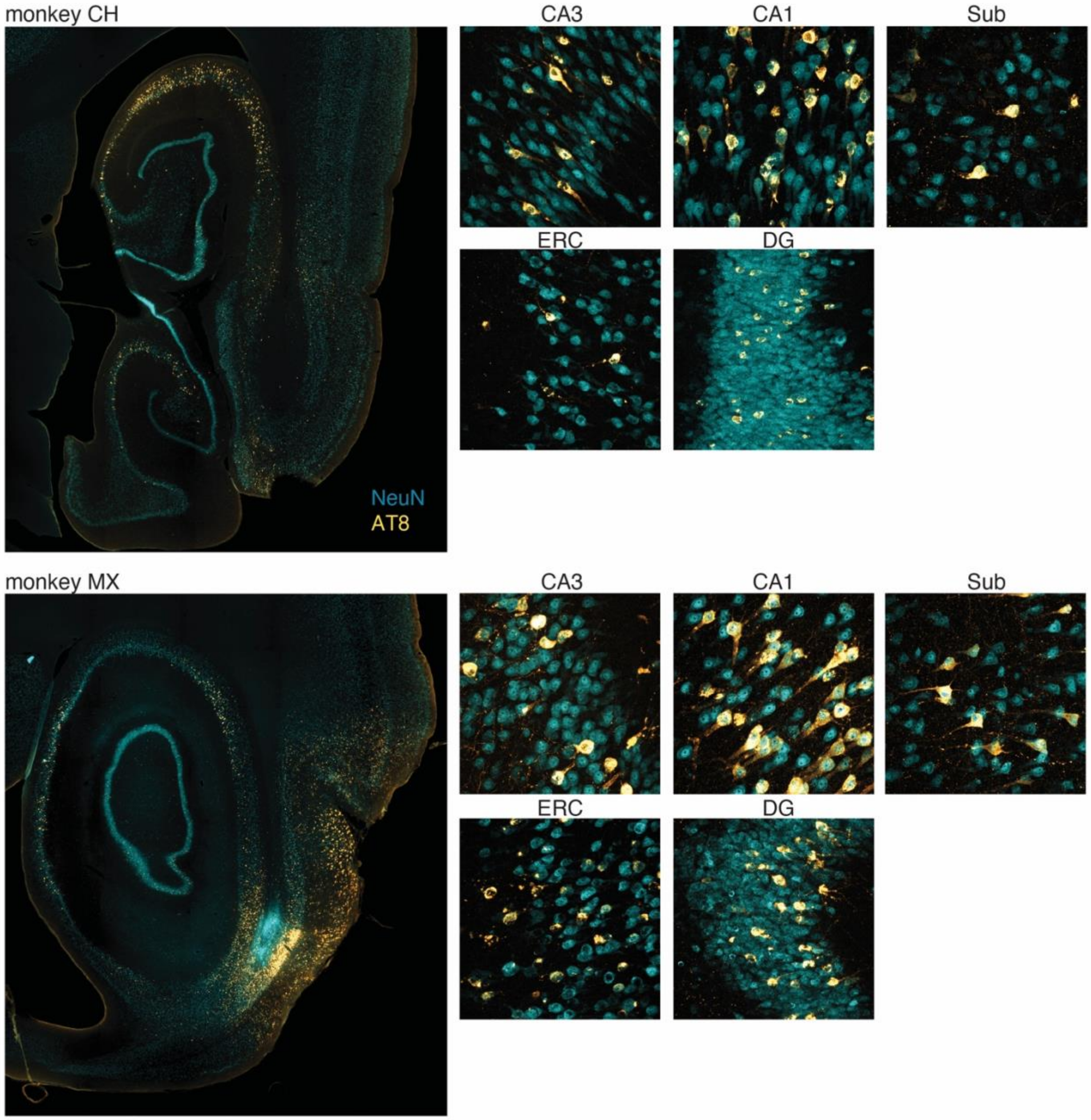

Supplementary Figure 1. AAV-2xTau injection in the entorhinal cortex (ERC) induces robust tau propagation. Histology performed one year after viral injection of dual tau mutation in the ERC. AT8 (gold) was used as a marker for pathological tau spread, with NeuN (blue) labelling neuronal nuclei. No AT8 labelling would be expected in these animals in the absence of the AAV-2xTau injection (see (17, 18)). Left hemisphere section shown for each animal. AAV injections were bilateral and tau was confirmed in both hemispheres. Increased magnification portions of the larger (left) image are shown for hippocampus (CA1 and CA3), subiculum (Sub), entorhinal cortex (ERC), and dentate gyrus (DG).

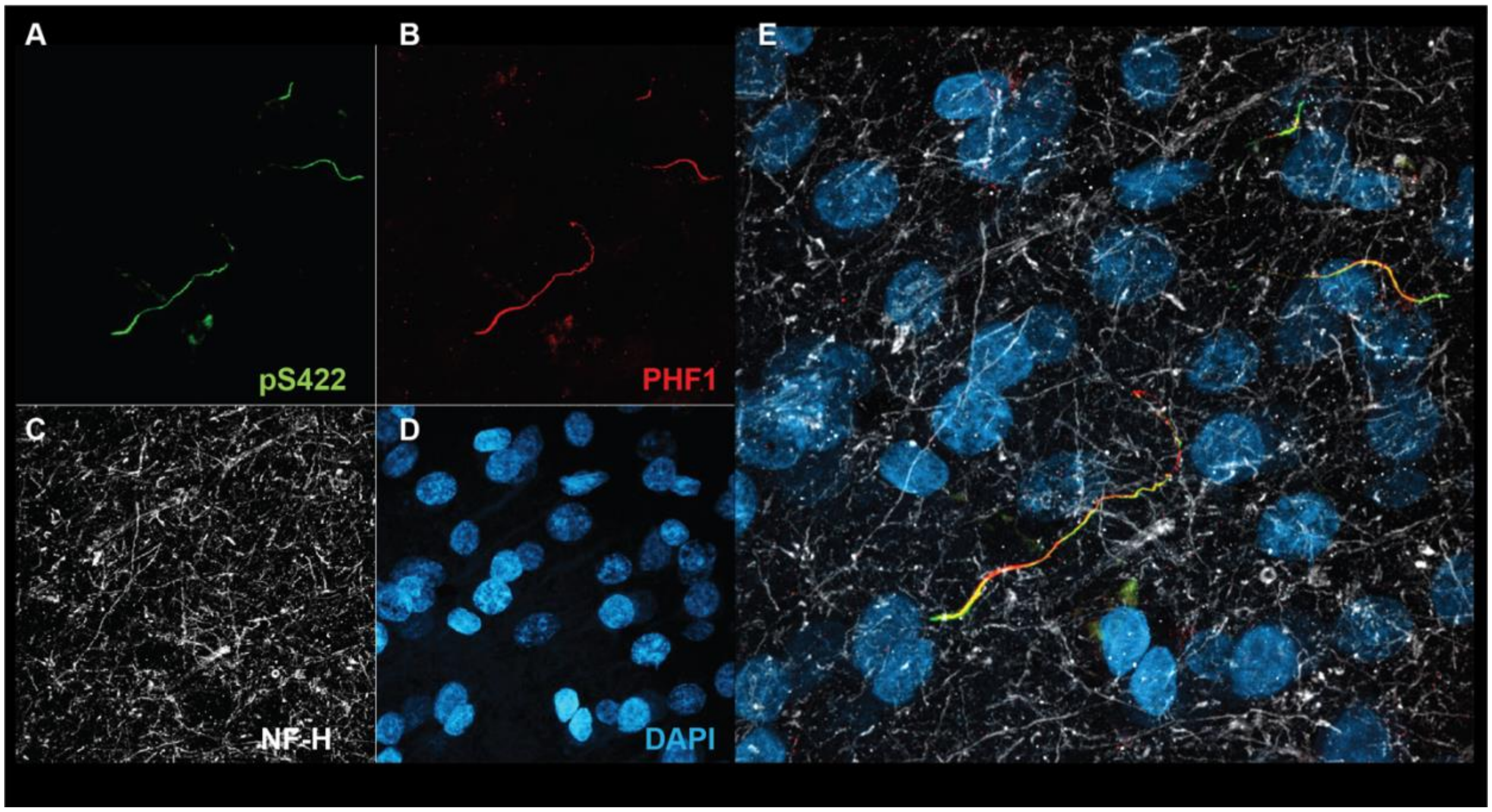

Supplementary Figure 2. Elaboration of histological observations in V4. The same field of view from Figure 1C is shown by compartment for tau markers A) pS422 and B) PHF1, C) Neurofilament Heavy, NF-H, which labels neuronal cytoskeletal elements, particularly in axons, and D) DAPI, which labels nuclei. E) shows panels A-D combined.
